# Enhancing fractalkine/CX3CR1 signalling pathway can reduce neuroinflammation by attenuating microglia activation in experimental diabetic retinopathy

**DOI:** 10.1111/jcmm.17179

**Published:** 2022-01-11

**Authors:** Mengmeng Jiang, Hai Xie, Chaoyang Zhang, Tianqin Wang, Haibin Tian, Lixia Lu, Jing‐Ying Xu, Guo‐Tong Xu, Lin Liu, Jingfa Zhang

**Affiliations:** ^1^ Department of Ophthalmology Renji Hospital Shanghai Jiao Tong University School of Medicine Shanghai China; ^2^ Tongji Eye Institute Tongji University School of Medicine Shanghai China; ^3^ Department of Ophthalmology Shanghai General Hospital (Shanghai First People’s Hospital) Shanghai Jiao Tong University Shanghai China; ^4^ National Clinical Research Center for Eye Diseases Shanghai Key Laboratory of Ocular Fundus Diseases Shanghai Engineering Center for Visual Science and Photomedicine Shanghai Engineering Center for Precise Diagnosis and Treatment of Eye Diseases Shanghai China

**Keywords:** diabetic retinopathy, fractalkine, microglia, neuroinflammation

## Abstract

The concept of diabetic retinopathy (DR) has been extended from microvascular disease to neurovascular disease in which microglia activation plays a remarkable role. Fractalkine (FKN)/CX3CR1 is reported to regulate microglia activation in central nervous system diseases. To characterize the effect of FKN on microglia activation in DR, we employed streptozotocin‐induced diabetic rats, glyoxal‐treated R28 cells and hypoxia‐treated BV2 cells to mimic diabetic conditions and explored retinal neuronal apoptosis, reactive oxygen species (ROS), as well as the expressions of FKN, Iba‐1, TSPO, NF‐κB, Nrf2 and inflammation‐related cytokines. The results showed that FKN expression declined with diabetes progression and in glyoxal‐treated R28 cells. Compared with normal control, retinal microglia activation and inflammatory factors surged in both diabetic rat retinas and hypoxia‐treated microglia, which was largely dampened by FKN. The NF‐κB and Nrf2 expressions and intracellular ROS were up‐regulated in hypoxia‐treated microglia compared with that in normoxia control, and FKN significantly inhibited NF‐κB activation, activated Nrf2 pathway and decreased intracellular ROS. In conclusion, the results demonstrated that FKN deactivated microglia via inhibiting NF‐κB pathway and activating Nrf2 pathway, thus to reduce the production of inflammation‐related cytokines and ROS, and protect the retina from diabetes insult.

## 1 INTRODUCTION

Diabetic retinopathy (DR), one of the common complications of diabetes mellitus, is the leading cause of blindness among working people worldwide.^1^ DR, traditionally considered as a microvascular disease, is also considered as neurodegeneration characterized by neuronal apoptosis and reactive gliosis.^2^, ^3^, ^4^, ^5^

Microglia, the resident monocytes of the retina, monitors the microenvironment of the retina and interact with other cells via numerous specific receptors.^6^ As the main immune cells in retina, microglia participates the pathogenesis of DR by morphological transition from the resting‐ramified morphology to activated‐amoeboid phenotype and releasing the inflammation‐related cytokines.^3^, ^4^, ^7^ It is widely reported that microglia activation results in microvascular damage, neurodegeneration and retinal inflammation,^4^, ^7^ leading to the dysfunction of neurovascular unit in retina.

Fractalkine (FKN), also known as CX3CL1, exists as two forms of ligands, that is the membrane‐bound and soluble forms. Both forms can bind the receptor CX3CR1. FKN is synthesized principally by neurons while CX3CR1 is merely expressed by microglia in retina.^8^, ^9^ A considerable amount of literatures shows that FKN/CX3CR1 signalling pathway, as an entry point of interaction between neurons and microglia, plays an important role in central nervous system diseases by modulating/inhibiting microglia activation.^10^, ^11^, ^12^, ^13^, ^14^, ^15^ In experimental DR, CX3CR1 deficiency potentiated retinal microglia changes,^16^ enhanced the inflammatory response as well as neuronal damage.^17^ In addition, CX3CR1 deficiency activated microglia, disrupted the vascular integrity and accelerated DR progression.^9^, ^18^ Furthermore, in mouse model of retinitis pigmentosa, FKN, delivered either by intravitreal injection or gene therapy, was protective for retinal neurons.^19^, ^20^ Based on the previous studies, we hypothesized that FKN administration might ameliorate microglia activation and proinflammatory cytokine release via CX3CR1 inhibitory signals in experimental DR, thus protecting the retina from diabetes insult by decreasing neuroinflammation.

To verify the hypothesis, we characterized the effect of FKN on microglia activation in experimental DR by employing streptozotocin‐induced diabetic rat, glyoxal‐treated R28 cells and hypoxia‐treated BV2 cells. The results showed that FKN treatment could ameliorate microglia activation via inhibiting NF‐κB pathway and enhancing Nrf2 pathway, thus to reduce the production of inflammation‐related cytokines and ROS, and protect the retina from diabetes insult.

## 2 MATERIALS AND METHODS

### 2.1 Reagents and antibodies

Recombinant rat fractalkine (*E. coli*‐derived chemokine domain of rat fractalkine protein, Gln25‐Gly100) was purchased from R&D (537‐FT‐025/CF, Shanghai, China). Streptozotocin (STZ, S0130) and glyoxal (50649) were purchased from Sigma‐Aldrich. The cell counting kit (CCK‐8, 40203ES60), protein extraction RIPA lysis buffer (PC101), protease inhibitor cocktail (20123ES10), phosphatase inhibitor cocktail (20109ES05), SYBR green real‐time PCR master mix (11123ES60) and reactive oxygen species assay kit (50101ES01) were purchased from Shanghai Yeasen Biotechnology Co. Ltd. DMEM (High Glucose, sh30243.01) and DMEM (Low Glucose, SH30021.01) were purchased from HyClone. Foetal bovine serum was purchased from Gibco (10091148, USA). Penicillin/streptomycin (15140155) was purchased from Invitrogen. Pierce BCA protein assay kit (23225) was purchased from Thermo Scientific. The information of the antibodies, used for Western blot and immunofluorescence, was provided in Table 1.

**TABLE 1:** List of antibodies used for Western blot and immunofluorescence

Antibody	Supplier	Catalogue#	Host	Dilution
Anti‐FKN	Abcam	Ab25088	Rabbit	1:500(WB)
Anti‐Iba−1	Abcam	ab178846	Rabbit	1:1,000(WB)
Anti‐Iba−1	Woka	019–19741	Rabbit	1:500(IF)
Anti‐TSPO	Abcam	Ab92291	Goat	1:1,000(WB) 1:500(IF)
Anti‐TNF‐α	SANTA	sc−52746	Mouse	1:500(WB)
Anti‐IL−1β	R&D Systems	AF−501‐SP	Goat	1:1,000(WB)
Anti‐ICAM−1	R&D Systems	AF583	Goat	1:1,000(WB)
Anti‐IL−6	R&D Systems	AF506‐SP	Goat	1:1,000(WB)
Anti‐p‐NFκB	CST	#3033	Rabbit	1:1,000(WB)
Anti‐NFκB	CST	#8241	Rabbit	1:1,000(WB) 1:500(IF)
Anti‐NRF2	CST	#12721	Rabbit	1:1,000(WB) 1:500(IF)
Anti‐β‐actin	CST	#3700	Mouse	1:5,000(WB)
Anti‐rabbit IgG (H+L) (Dy Light™ 680)	CST	#5366	Goat	1:1000(WB)
Anti‐mouse IgG (H+L) (Dy Light™ 800)	CST	#5257	Goat	1:1000(WB)
Anti‐Goat IgG (H+L) (IRD ye^®^ 800)	Abcam	ab216775	Donkey	1:1000(WB)
Anti‐Rabbit IgG(HRP)	Yeasen	34201ES60	Donkey	1:1000(WB)
Anti‐Mouse IgG (HRP)	Yeasen	34101ES60	Donkey	1:1000(WB)
Anti‐Rabbit IgG H&L (Alexa Fluor^®^ 647)	Abcam	ab181347	Donkey	1:500(IF)
Anti‐Rabbit IgG H&L (Alexa Fluor^®^ 488)	Abcam	ab150061	Donkey	1:500(IF)
Anti‐Goat IgG H&L (Alexa Fluor^®^ 555)	Abcam	ab150134	Donkey	1:500(IF)
Isolectin GS‐IB4	Invitrogen	I21411	‐	1:2,000(IF)

Abbreviations: CST, cell signalling technology; IF, immunofluorescence; WB, Western blot.

### 2.2 Experimental animals

The Sprague‐Dawley (SD) rats were treated in agreement with the ARVO statement for Use of Animals in Ophthalmic and Vision Research and The Guides for the Care and Use of Animals (National Research Council). The protocol was approved by the ethics committee of Animal Experiments of Tongji University (Permit Number: TJHBLAC‐2020–06). Male Sprague‐Dawley rats with body weight 120–160 g were purchased from Slaccas, maintained under 12‐hour light/dark cycle and were given *ad libitum* access to food and water.

To induce diabetes, the rats were injected intraperitoneally with STZ (60 mg/kg BW in citric acid buffer) after fasted for 24 h, while the normal control received an equal volume of citric acid buffer according to our previous study.^21^ Blood glucose level was measured by glucometer for three consecutive days, and rats with blood glucose levels more than 16.6 mmol/L were included and considered as diabetic rats. Two hours after STZ injection, the right eyes of diabetic rats were injected intravitreally with recombinant FKN protein (0.2 mg/eye, 2 μl) as D+F group, while the left eyes were received the equivalent volume of phosphate‐buffered saline (PBS) as D group. The age‐matched normal control was injected intravitreally with equivalent volume of PBS and designated as N group. Rats were sacrificed at 4 to 12 weeks of diabetes.

### 2.3 Retinal sample preparation

The rats were sacrificed with cervical dislocation after complete anaesthesia. Both eyes were enucleated and immediately fixed in 4% PBS‐buffered paraformaldehyde overnight at 4℃.

For cryosection, the eyecups were dehydrated through a gradient concentration of sucrose from 10% to 30%. In addition, then, the eyecups were embedded in OCT compound (Tissue Tek; Sakura). Frozen serial sections (10 μm thickness) were prepared on a cryostat microtome (Leica).

To prepare retinal flatmount, the neurosensory retina was isolated carefully and flattened on microscope slide with four radial cuts.

### 2.4 Cell cultures

R28 cells, an E1A immortalized model of retinal neurons, were a generous gift from Gail M. Seigel (State University of New York). The R28 cells were cultured in DMEM with low glucose (5 mM), 10% fetal bovine serum and 1% penicillin/streptomycin at 37°C under 5% CO_2_ in a humidified incubator. R28 cells were plated for 48 h and then divided into normal control group (N) and glyoxal‐treated group (G).

BV2 cells, a mouse microglial cell line, were purchased from American Type Culture Collection and cultured in DMEM with high glucose (25 mM), 10% fetal bovine serum and 1% penicillin/streptomycin at 37°C under 5% CO_2_ in a humidified incubator. After 24 h culture, the cells were divided into three groups: normal control (N), hypoxia treatment (H) and hypoxia+FKN treatment (H+F). The latter two groups were incubated in a hypoxic workstation (Whitley H35 Hypoxystation; Don Whitley Scientific, Bingley, UK) with 1% O_2_ with or without FKN treatment.

### 2.5 Cell death detection in retinal cryosection and R28 cells by TUNEL Assay

The TUNEL assay was performed on retinal cryosections and R28 cells with *In Situ* Cell Death Detection Kit (Roche China, Ltd.). Positive controls were retinal sections or R28 cells that had been treated with grade I DNase‐I for 20 minutes at room temperature before the labelling procedure. Negative controls were the retinal sections or R28 cells only treated with 50 μl label solution in absence of enzyme solution. After being incubated and washed three times with PBS, the sections were observed under fluorescence microscope (Nikon), with an excitation wavelength in the range of 450 to 490 nm.

### 2.6 Immunofluorescence

For immunostaining in retinal flatmount, the retinas were incubated with membrane penetrating solution (2.5% Triton X‐100 and 2.5%Tween‐20 in 1 × PBS) overnight on a rocking platform overnight at 4°C and then incubated in blocking solution (1% W/V nonfat dry milk, 0.1% Tween‐20 in 1 × PBS) for 4 h at room temperature or overnight at 4°C. After being incubated with anti‐Iba‐1 rabbit polyclonal antibody to label microglia overnight at 4°C, the retinas were washed three times with 1 × PBS and then incubated with secondary antibody (goat anti‐rabbit IgG Alexa Fluor 647 conjugate) and isolectin GS‐IB4 to label retinal vasculature (Alexa Fluor 488 conjugate) for 2 h at room temperature. After being washed three times with 1 × PBS, the retinal flatmounts were cover‐slipped and examined under a confocal microscope.

For immunostaining in BV2 cells, BV2 cells were fixed with 4% paraformaldehyde for 30 min at room temperature and then incubated with the blocking solution (1% BSA/0.05% Triton X‐100 in 1×PBS) for 1  hour at room temperature. After that, cells were incubated with rabbit anti‐p65 primary antibody or rabbit anti‐Nrf2 primary antibody, respectively, overnight at 4°C, followed by Alexa Fluor 647‐conjugated or 488 conjugated secondary antibody for 2 h at room temperature. After washed three times, the coverslips were mounted on microscope slides with 20 μl DAPI‐Fluoromount‐G (0100–20; Southern Biotech) to stain nuclei. The fluorescent images were observed and captured with a confocal laser‐scanning microscope (Nikon). For co‐localization analysis, fluorescence intensity through the nucleus of single cells was profiled using ImageJ software (Rawak Software Inc., Stuttgart, Germany) and be visualized by graphs drawn with GraphPad Prism Software, version 7.0 (GraphPad Software, Inc.).

### 2.7 Cell viability assay

The cell viability was detected by Cell Counting Kit‐8. The R28 cells were seeded on 96‐well plates at a density of 1.0 × 10^4^ cells per well and incubated in the medium containing glyoxal (0, 0.05, 0.1, 0.5, 1, 2, 2.5, 5 and 10 mM) for 12 h to determine the optimal dose of glyoxal on R28 cells. CCK‐8 solution was added in a ratio of 1:10 with final concentration at 110 μl/ well. Two hours later, the cell viability was measured with the absorbance at 450 nm using a microplate spectrophotometer (Tecan, Crailsheim, Germany).

### 2.8 Protein extraction and western blot

The samples, including the retinas, R28 cells and BV2 cells, were lysed in RIPA buffer supplemented with protease inhibitor cocktail and phosphatase inhibitor cocktail, and sonicated for 15 s and then placed on ice for 30 min. Protein concentrations were determined with BCA protein assay kit (Thermo Scientific). Equivalent amounts of proteins were resolved on SDS‐polyacrylamide gels (10~15%) and transferred onto nitrocellulose membranes (Bio‐Rad). The membranes were cut according to the molecular weight of each protein. After being blocked with 5% BSA (Thermo Scientific) in PBS at room temperature for 30 min, membranes were separately incubated with the primary antibodies over night at 4°C. After being washed for three times with PBS‐buffered Tween‐20 (PBST), the membranes were incubated with the corresponding secondary antibodies at room temperature for 2 h, followed by washes with PBST for three times and then imaged by chemiluminescence or Odyssey infrared imaging system (LI‐COR Biosciences). The optical density of each band was quantified by ImageJ software, and the densitometric values for the proteins were normalized by β‐actin.

### 2.9 Quantitative RT‐PCR

Total RNA was extracted from BV2 cells in 6‐well plates. The reverse transcription product was examined by real‐time PCR. The specific primers were designed online through PUBMED website and purchased from manufacturer (Biotechnology Corporation Ltd. Shanghai, China). The primers for TNF‐α, IL‐1β, ICAM‐1, IL‐6 and β‐actin are shown in Table 2. Real‐time PCR was performed in a 20 µl system containing SYBR green real‐time PCR master mix (10 µl), ddH_2_O (6 µl), primers (0.5 µM each, 1 µl for each primer) and cDNA (2 µl). All reactions were performed in Real‐Time PCR System connected CFX Manager (Bio‐Rad, USA). The relative gene expressions were normalized by β‐actin.

**TABLE 2:** Primer information for real‐time PCR

Gene	Forward Primer (5′−3′)	Reverse Primer (5′−3′)
β‐actin	AGGCGACAGCAGTTGGTTGGA	TTGGGAGGGTGAGGGACTTCCT
TNFα	GCCACCACGCTCTTCTGTCTAC	AGTGTGAGGGTCTGGGCCATAG
ICAM−1	GCTCGGAGGATCACAAACGA	AGTCTGCTGAGACCCCTCTT
IL−1β	CTTATTACAGTGGCAATGAGGATG	CTTTCAACACGCAGGACAGGTACA
IL−6	GCTACCAAACTGGATATAATCAGGA	CCAGGTAGCTATGGTACTCCAGAA

### 2.10 Detection of Intracellular ROS Level

Intracellular ROS level was detected by Reactive Oxygen Species Assay Kit according to the manufacturer's instructions. Cells with approximately 50~60% confluence were incubated for 3 h and stained with DCFH‐DA (10 mM diluted by serum‐free cell culture medium) for 30 min, while negative control was only incubated with serum‐free cell culture medium. The cells were washed with serum‐free culture medium for two times to fully remove free DCFH‐DA. The fluorescent images were captured under fluorescence microscope (Nikon, Yokohama, Japan) with an excitation wavelength in the range of 450 to 490 nm. To quantify the DCF fluorescence, the cells were collected and assayed with flow cytometry at Ex/Em: 485/535 nm signal on BD LSRFortessa (Becton, Dickinson and Company, NJ, USA). The results were processed and plotted with FCS Express 7 Demonstration (De Novo Software).

### 2.11 Statistical analysis

All experiments were repeated at least three times. Data were expressed as mean ± SE. The statistical analysis was carried out by using 2‐tailed Student's *t* test; a *P* value of 0.05 or less was considered statistically significant.

## 3 RESULTS:

### 3.1 Establishment of the diabetic rat model

**FIGURE 1:**
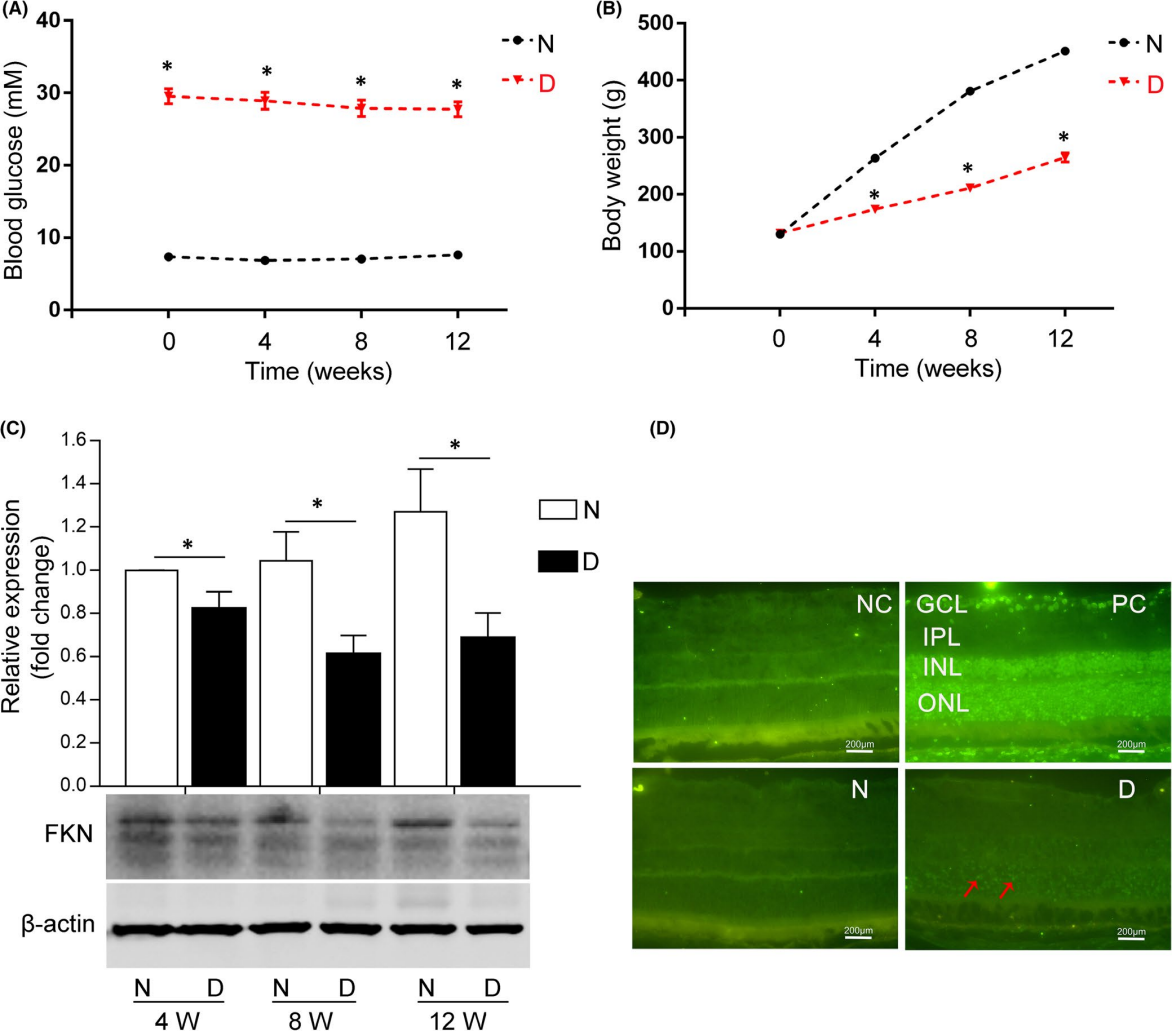
FKN expression declined with diabetes progression accompanied with cell apoptosis in STZ‐induced diabetic rat retina. (A) The changes of blood glucose level in diabetic rats with diabetes progression. (B) The changes of body weight in diabetic rats with diabetes progression. (C) Retinal FKN expression was down‐regulated with diabetes progression in diabetic rats detected by Western blot and analysed with semi‐quantification. (D) Retinal cell apoptosis was detected by TUNEL assay (red arrows indicated TUNEL‐positive apoptotic cells). Data were expressed as mean ± SE (*n* = 8 rats per group in [A] and [B], and *n* = 9 retinas per group in [C], *p < 0.05 compared with age‐matched normal group in [A], [B] and [C]). N, normal control; D, diabetic group; NC, negative control; PC, positive control; 4 W, 4 weeks after diabetes onset; GCL, ganglion cell layer; IPL, inner plexiform layer; INL, inner nuclear layer; ONL, outer nuclear layer. Scale bar: 200 μm

Diabetic rat model was established by intraperitoneal injection of STZ. After diabetes onset, over a 12‐week period, blood glucose levels in the diabetic group increased significantly compared with the control condition (Figure 1A), that is 29.55 ± 1.05 mM vs. 7.36 ± 0.39 mM (day 1), 28.93 ± 1.17 mM vs. 6.84 ± 0.45 mM (week 4), 27.88 ± 1.15 mM vs. 7.06 ± 0.38 mM (week 8) and 27.74 ± 1.05 mM vs. 7.63 ± 0.53 mM (week 12), respectively, (n =8 in normal control and n =8 in diabetic rats).

The body weight (BW) of both the nondiabetic control and diabetic rats increased with time. But compared with the normal control, the BW of the diabetic rats decreased significantly (Figure 1B), that is decreased by 40.8% (week 4), 44.7% (week 8) and 41.3% (week 12) respectively, (*n* = 8 in normal control and *n* = 8 in diabetic rats). The elevated blood glucose level and the decreased BW compared with normal control indicated the successful establishment of diabetic rat model.

### 3.2 FKN expression declined with diabetes progression and the neuronal apoptosis increased in diabetic rat retinas

To detect the expression of FKN and apoptosis of retinal cells in experimental DR, we performed Western blot assay and TUNEL assay in diabetic rat retinas. Western blot densitometry analysis results showed the protein expression of FKN decreased significantly compared with that in normal control, that is decreased by 17.8% (4 weeks, *n* = 9, *p*=0.038), 41.4% (8 weeks, *n* = 9, *p =* 0.016) and 46.0% (12 weeks, *n* = 9, *p* = 0.022) respectively (Figure 1C). We also detected the retinal cell apoptosis at week 4 after diabetes onset, and the result showed that apoptosis, especially in outer nuclear layer (ONL), increased compared with that in normal control (Figure 1D).

### 3.3 FKN expression declined and the apoptosis increased in glyoxal‐treated R28 cells

**FIGURE 2:**
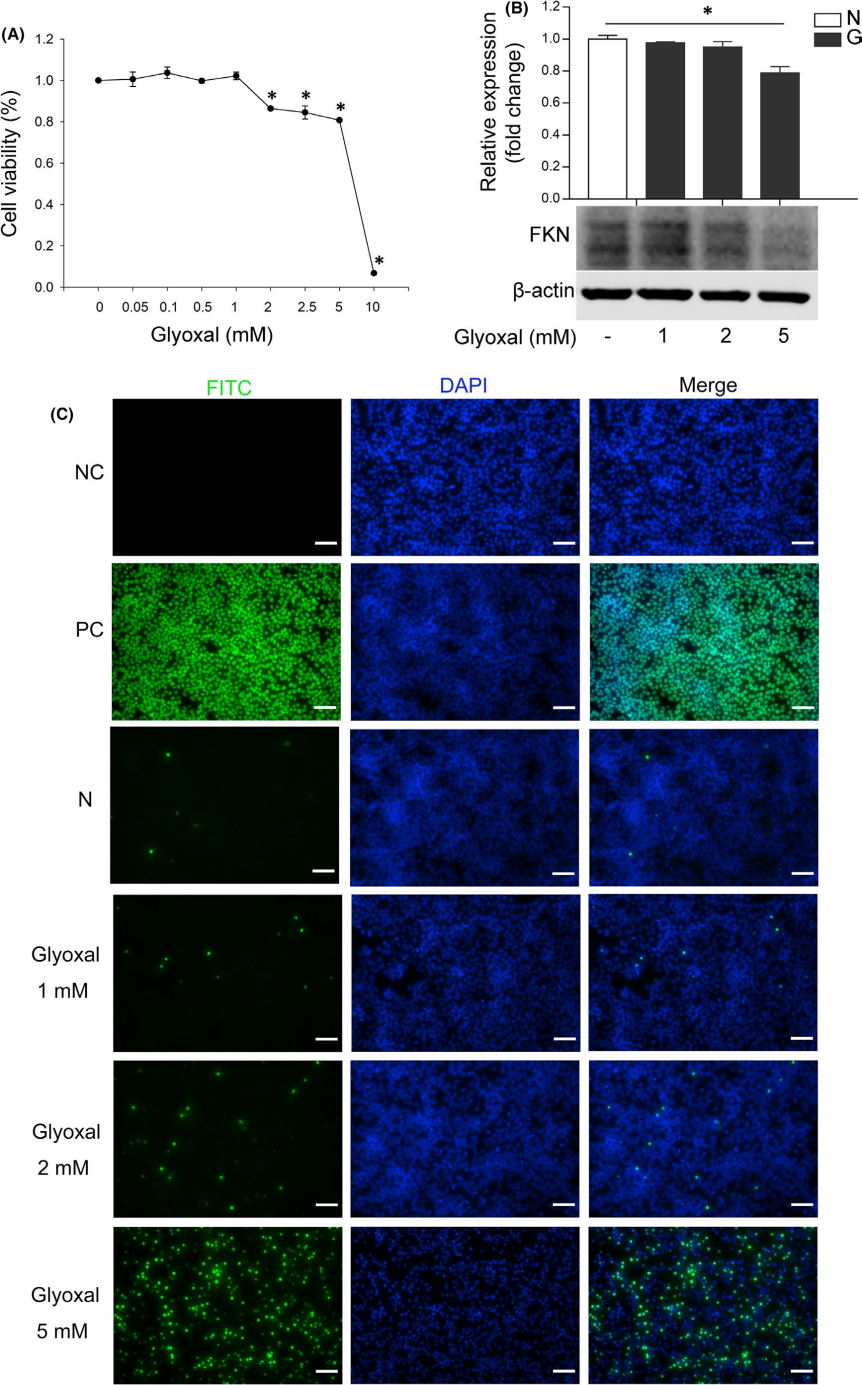
FKN expression declined with cell apoptosis in glyoxal‐treated R28 cells. (A) Cell viability assay of the R28 cells treated with different concentrations of glyoxal for 12 h. (B) Western blot and densitometry analysis showed the expression of FKN was decreased dose‐dependently in R28 cells treated with different doses of glyoxal for 12 h. (C) TUNEL assay of R28 cells treated with different doses of glyoxal for 12 h. Data were expressed as mean ± SE (*n* = 4 samples per group in [A], n = 3 samples per group in [B]), and *p < 0.05 compared with the control group without glyoxal treatment in [A] and [B]. FKN, Fractalkine; N, normal control; G, glyoxal; NC, negative control; PC, positive control. Scale bar: 35 μm

Since neurons are the main source for FKN,^22^, ^23^ we speculated that decreased FKN expression in diabetic retina might be due to the neuronal apoptosis. To confirm this, we used R28 cells and treated R28 cells with glyoxal as an *in vitro* model to mimic diabetic condition. The cell viability of R28 cells was decreased in a dose‐dependent manner after 12 h incubation with different doses of glyoxal (Figure 2A). For example, the cell viability was decreased by 13.6% (2 mM, *p* < 0.0001), 19.2% (5 mM, *p* < 0.0001) and 93.2% (10 mM, *p* < 0.0001) respectively. Based on this result, we treated R28 cells with glyoxal at 1, 2 and 5 mM for 12 h and examined FKN protein expression and cell apoptosis. As shown in Figure 2B, the protein expression of FKN was decreased by 2.4% (1 mM, *p* = 0.35), 5.0% (2 mM, *p* = 0.25) and 21.1% (5 mM, *p* = 0.003) respectively. In the meantime, the R28 cell apoptosis was elevated with the increasing glyoxal concentration (Figure 2C). These results indicated that the decreased FKN expression in diabetic rat retinas might be associated with the loss of retinal neurons (Figures 1, 2).

### 3.4 FKN attenuated retinal microglia activation in diabetic rats

**FIGURE 3:**
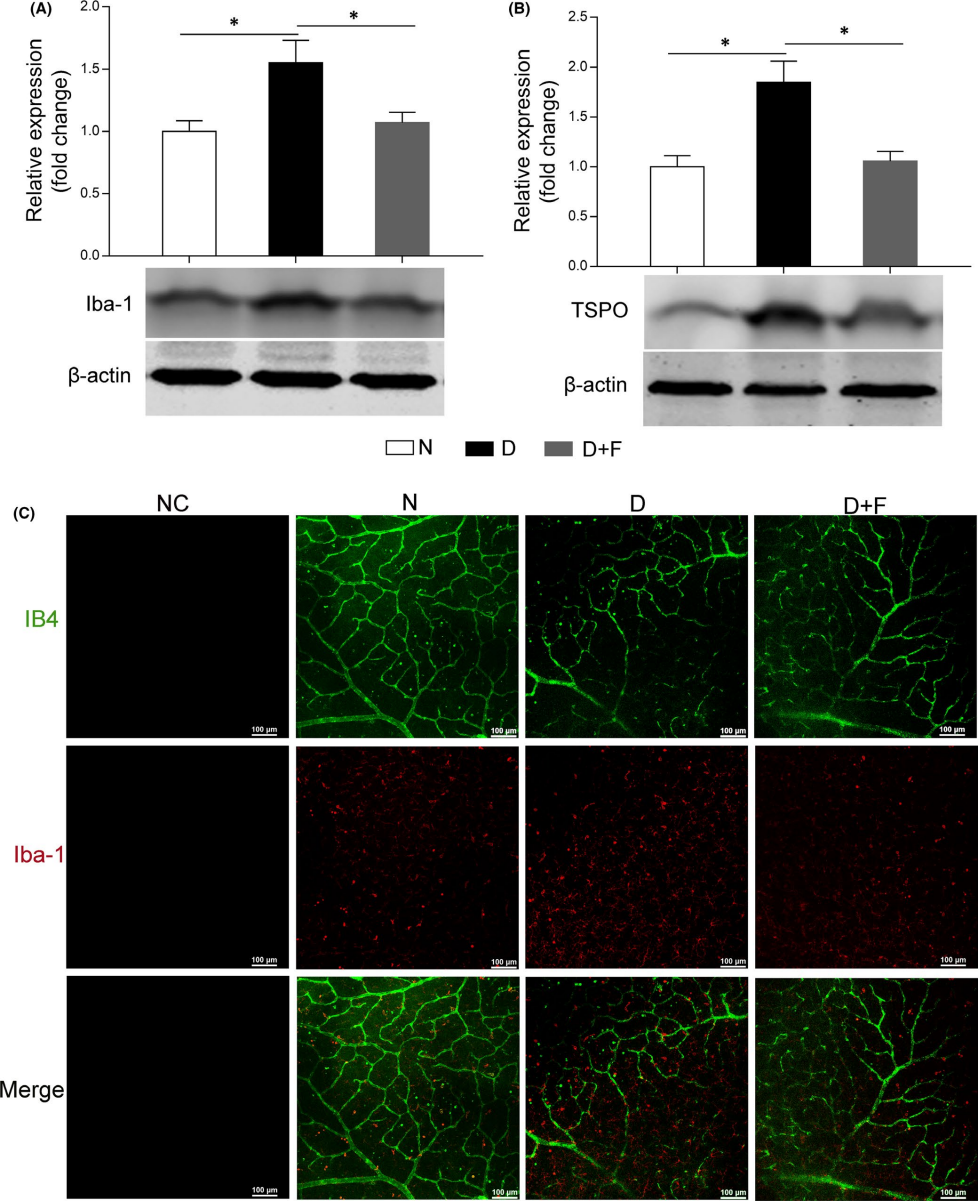
Intravitreal injection of FKN attenuated microglia activation in diabetic rat retina. (A and B) Western blot densitometry analysis of retinal microglia markers Iba‐1 (A) and TSPO (B). (C) Immunofluorescence with IB4 (green) and Iba‐1 (red) in retinal flatmount. Data were expressed as Mean ± SE (n = 8 retinas per group), and *p < 0.05 compared with diabetes group. N, normal control; D, diabetic group; D+F, diabetic rats treated with intravitreal injection of 0.2 mg (0.1 mg/μl, 2 μl) recombinant FKN. Scale bar: 100 μm

Considering that the FKN receptor (CX3CR1) is uniquely expressed in microglia, which is activated in early DR,^3^, ^7^ the lack of inhibitory effect on CX3CR1 due to decreased FNK might activate microglia in diabetic rat retinas. We explored the effect of exogenous FKN on retinal microglia activation after intravitreal injection. According to Western blot densitometry analysis results, compared with that in normal control, the protein expressions of Iba‐1 and TSPO (microglia markers) increased significantly by 55.3% (*n* = 8, *p* = 0.014, Figure 3A) and 84.7% (*n* = 8, *p* = 0.0033, Figure 3B). The surge of Iba‐1 and TSPO in diabetic rat retinas was decreased by 31.0% (*n* = 8, *p* = 0.029, Figure 3A) and 42.7% (*n* = 8, *p* = 0.0045, Figure 3B) following FKN exposure. To further confirm the effect of FKN on microglia, we performed Iba‐1 immunostaining on the retinal flatmount. As shown in Figure 3C, compared with the normal control, the microglia became activated in diabetic rat retinas with increasing number and more amoeboid morphologies, which was largely abolished by FKN. The above results indicated the inhibitory effect of FKN on microglia activation might be via CX3CR1.

### 3.5 FKN treatment reduced retinal inflammation in diabetic rats

**FIGURE 4:**
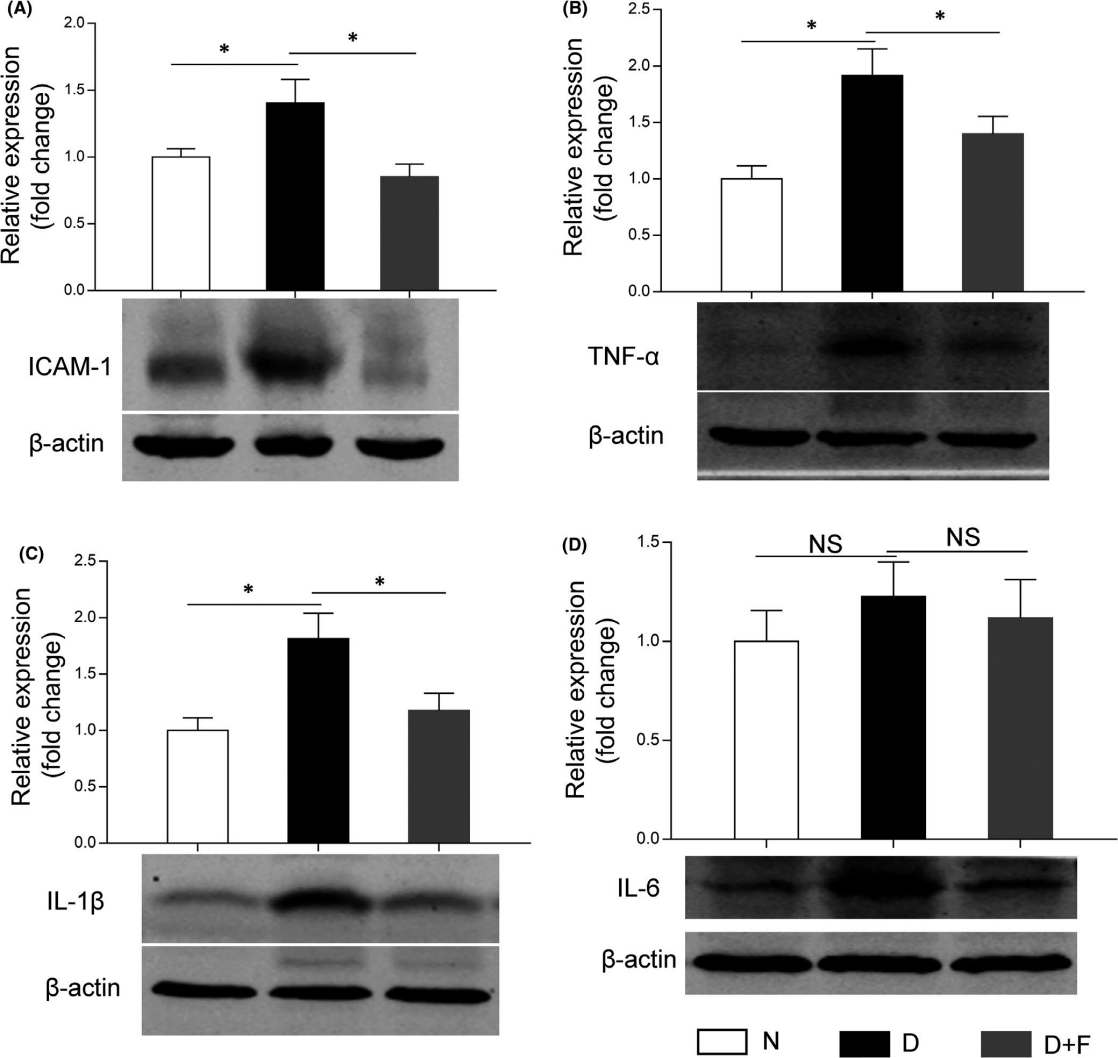
Intravitreal injection of FKN reduced the expression of inflammation‐related cytokines in diabetic rat retina. (A, B, C, D) Western blot densitometry analysis of the expression levels of ICAM‐1, TNF‐α, IL‐1β and IL‐6 in diabetic rat retinas with or without FKN treatment. Data were expressed as Mean ± SE (*n* = 9 retinas per group in [A] and [B], *n* = 8 retinas per group in [C] and [D]) and *p < 0.05 compared with diabetes group, NS, no significant difference). N, normal control; D, diabetes; D+F, diabetic rats treated with intravitreal injection of 0.2 mg (0.1 mg/μl, 2 μl) recombinant FKN

Microglia activation and its secretion of inflammation‐related cytokines are important components of neuroinflammation in DR.^3^, ^7^ Meanwhile, the FKN/CX3CR1 signalling pathway, involved in the inflammatory networks,^23^, ^24^ inspired us to study the effect of exogenous FKN treatment on retinal inflammation in DR. As Western blot densitometry analysis results shown in Figure 4, compared with that in normal control, the inflammation‐related cytokines, including ICAM‐1, TNF‐α and IL‐1β increased significantly in diabetic group, that is increased by 40.4% (ICAM‐1, *n* = 8, *p* = 0.047), 91.5% (TNF‐α, *n* = 9, *p* = 0.00057) and 81.4% (IL‐1β, *n* = 9, *p* = 0.0053) respectively. Interestingly, we observed FKN significantly down‐regulated these inflammation‐related cytokines, that is decreased ICAM‐1 by 39.4% (*n* = 8, *p* = 0.015), TNF‐α by 26.9% (*n* = 9, *p =* 0.026) and IL‐1β by 35.0% (*n* = 9, *p* = 0.034). Although the expression of IL‐6 showed the same trend as above cytokines, that is increased by 22.6% in diabetic group and decreased by 8.8% following FKN treatment, there was no significant difference among these groups.

### 3.6 FKN attenuated hypoxia‐induced microglia activation and inflammation‐related cytokines in vitro

**FIGURE 5:**
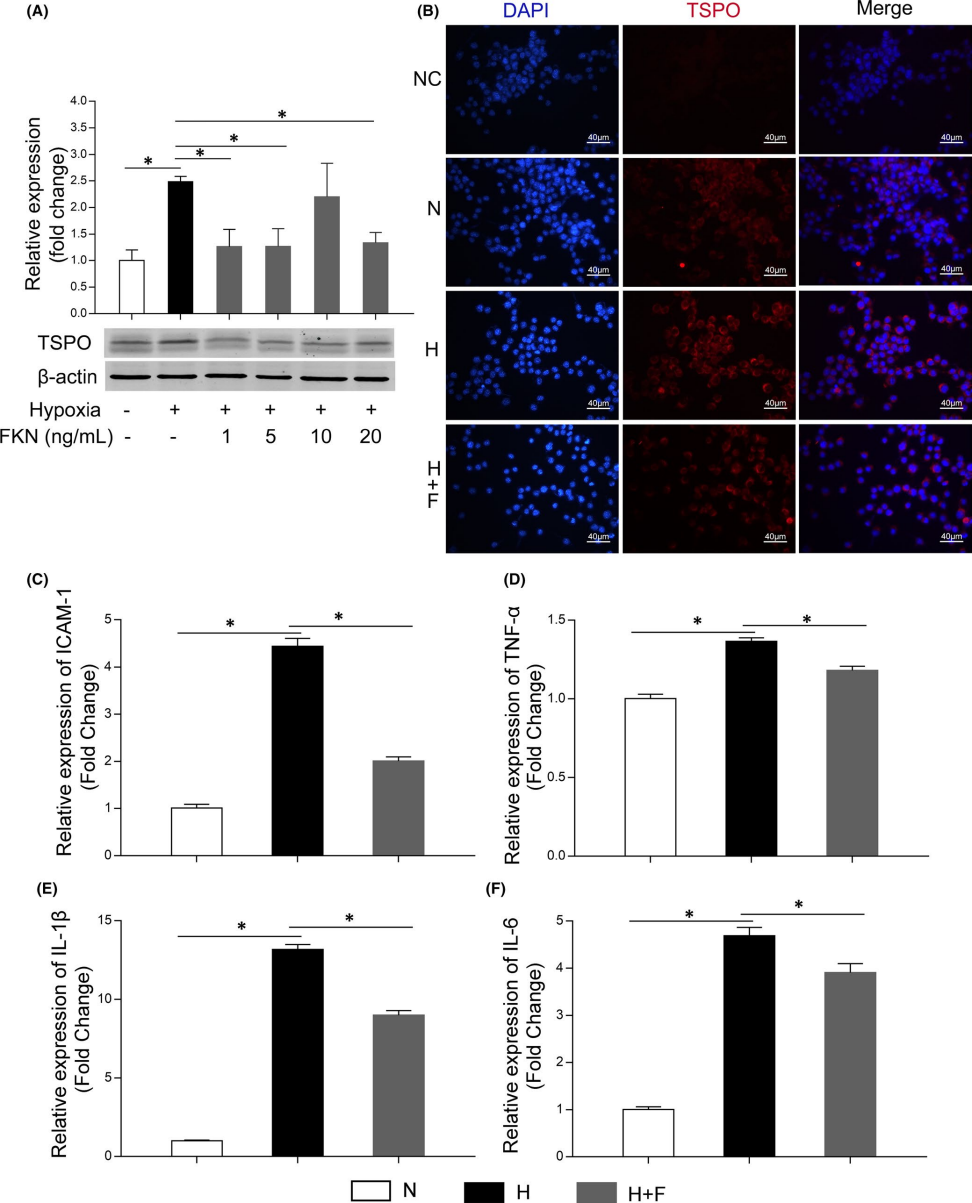
FKN attenuated hypoxia‐induced microglia activation and decreased mRNA expressions of inflammation‐related cytokines in BV2 cells. (A) Western blot densitometry analysis of TSPO (microglia activation marker) expression in hypoxia‐treated BV2 cells with or without FKN treatment. (B) Immunofluorescence of TSPO (red) and DAPI (blue) in hypoxia‐treated BV2 cells with or without FKN treatment. (C‐F) The mRNA expressions of pro‐inflammatory cytokines (ICAM‐1, TNF‐α, IL‐1β and IL‐6) in hypoxia‐treated BV2 cells with or without FKN treatment. Data were expressed as mean ± SE (*n* = 4 samples per group), *p < 0.05 compared with hypoxic group. N, normal control; H, Hypoxic group; H+F, hypoxia‐treated BV2 cells co‐treated 5 ng/ml FKN. Scale bar: 40 μm

To confirm the effect of FKN on microglia in diabetic rat retinas, we adopted hypoxia‐treated BV2 cells as an *in vitro* model and treated BV2 cells with or without FKN. According to Western blot densitometry analysis results, the protein expression of TSPO was increased significantly under hypoxia compared with that in normoxia, which was then down‐regulated by FKN (Figure 5A). Compared with that in normal control, the protein expression of TSPO was increased by 148.6% (*n* = 4, *p* = 0.00058) and decreased by 49.1% (1 mM, *n* = 4, *p* = 0.011), 49.0% (5 mM, *n* = 4, *p* = 0.013) and 46.2% (20 mM, *n* = 4, *p* = 0.0020), respectively, with FKN treatment. The protein changes of TSPO were also confirmed with immunofluorescence, the fluorescent intensity of TSPO was much stronger in hypoxic group compared with normal control, which was decreased by FKN treatment (Figure 5B).

To detect the effect of FKN on the expression of inflammation‐related cytokines in hypoxia‐treated BV2 cells, we performed Real‐Time Quantitative PCR to evaluate the mRNA levels of inflammation‐related cytokines. As shown in Figure 5C‐F, compared with the control group, the mRNA levels of ICAM‐1, TNF‐α, IL‐1β and IL‐6 all increased significantly, that is increased by 342.8% (ICAM‐1, *n* = 4, *p *< 0.0001), 36.5% (TNF‐α, *n* = 4, *p* < 0.0001), 1216.4% (IL‐1β, n = 4, *p* < 0.0001) and 368.2% (IL‐6, *n* = 4, *p* < 0.0001), respectively, in hypoxic group. FKN treatment could decrease significantly the mRNA levels of these inflammation‐related cytokines, that is decreased by 54.7% (ICAM‐1, *n* = 4, *p *< 0.0001), 13.5% (TNF‐α, *n* = 4, *p* = 0.0020), 31.6% (IL‐1β, *n* = 4, *p *< 0.0001) and 16.7% (IL‐6, *n* = 4, *p* = 0.024) respectively.

### 3.7 FKN suppressed NF‐κB activation in hypoxia‐induced microglia

To study the molecular mechanisms by which the FKN suppressed the inflammatory factors in microglia, we detected the activation of the transcription factor NF‐κB, which is wildly reported to be related to microglia‐associated neuroinflammation.^7^, ^25^

**FIGURE 6:**
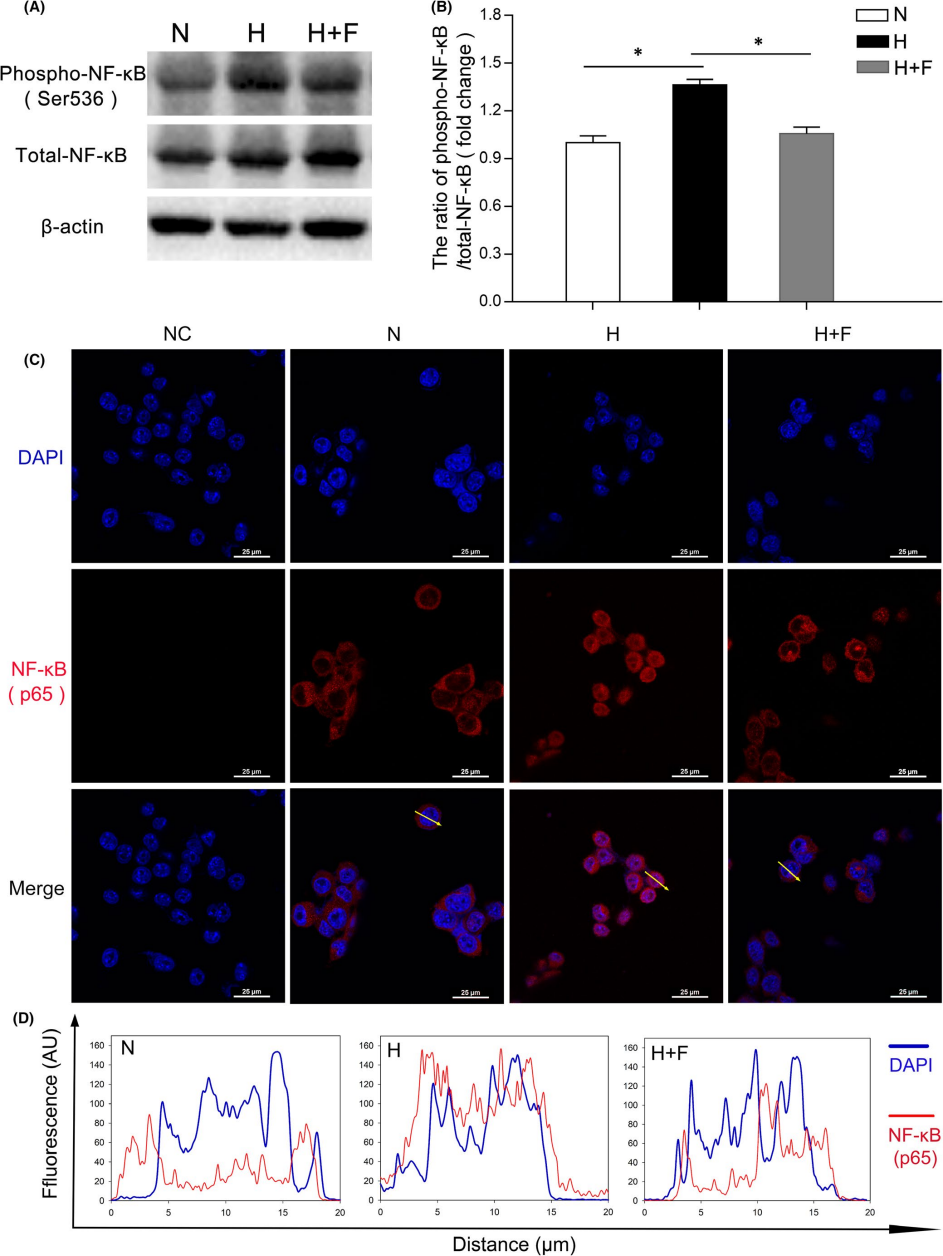
FKN suppressed NF‐κB activation in hypoxia‐induced microglia. (A and B) Western blot densitometry analysis of phosphor‐NF‐κB and NF‐κB in hypoxia‐treated BV2 cells with or without FKN treatment. (C) Immunofluorescence of NF‐κB in hypoxia‐treated BV2 cells with or without FKN treatment. (D) The quantitation of the fluorescence intensity among three groups. The profiles along an ideal oriented line across a representative cell indicated by yellow arrows in [C] was quantitated by ImageJ software. Data were expressed as mean ± SE (*n* = 8 samples per group, *p < 0.05 compared with hypoxia group). AU, Arbitrary Units. N, normal control; H, Hypoxic group; H+F, hypoxia‐treated BV2 cells co‐treated 5 ng/ml FKN. Scale bar: 25 μm

Compared with the normal control, the Western blot densitometry analysis result showed the ratio of phospho‐NF‐κB p65 to total NF‐κB p65 was increased significantly in hypoxia group, increased by 36.4% (*n* = 8, *p *< 0.0001), which was decreased significantly by 22.6% (*n* = 8, *p *< 0.0001) after FKN treatment (Figure 6A‐B). To further characterize the activation of NF‐κB, we performed immunostaining and monitored the translocation of NF‐κB to the nucleus in BV2 cells. As shown in Figure 6C and Figure 6D, NF‐κB was mainly distributed in cytoplasm in normal control group, while hypoxia induced a significant translocation of NF‐κB from the cytoplasm to the nucleus, indicating the enhancing transcriptional activity for proinflammatory factors. Interestingly, we were able to observe that FKN treatment could largely inhibit the translocation of NF‐κB.

### 3.8 FKN enhanced the activation of Nrf2 and elimination of ROS in hypoxia‐induced microglia

Nrf2 is a transcription factor which enters the cell nucleus to promote the expression of antioxidant‐response genes leading to elimination of ROS^22^, ^26^, ^27^, ^28^ in addition to its anti‐inflammation effects.^29^, ^30^ Moreover, previous studies suggested that Nrf2 may be a relevant downstream target of FKN to limit microglial over‐activation and decrease neuroinflammation.^26^, ^31^ Hence, we detected the changes of Nrf2 and ROS production in hypoxia‐treated microglia with or without FKN.

**FIGURE 7:**
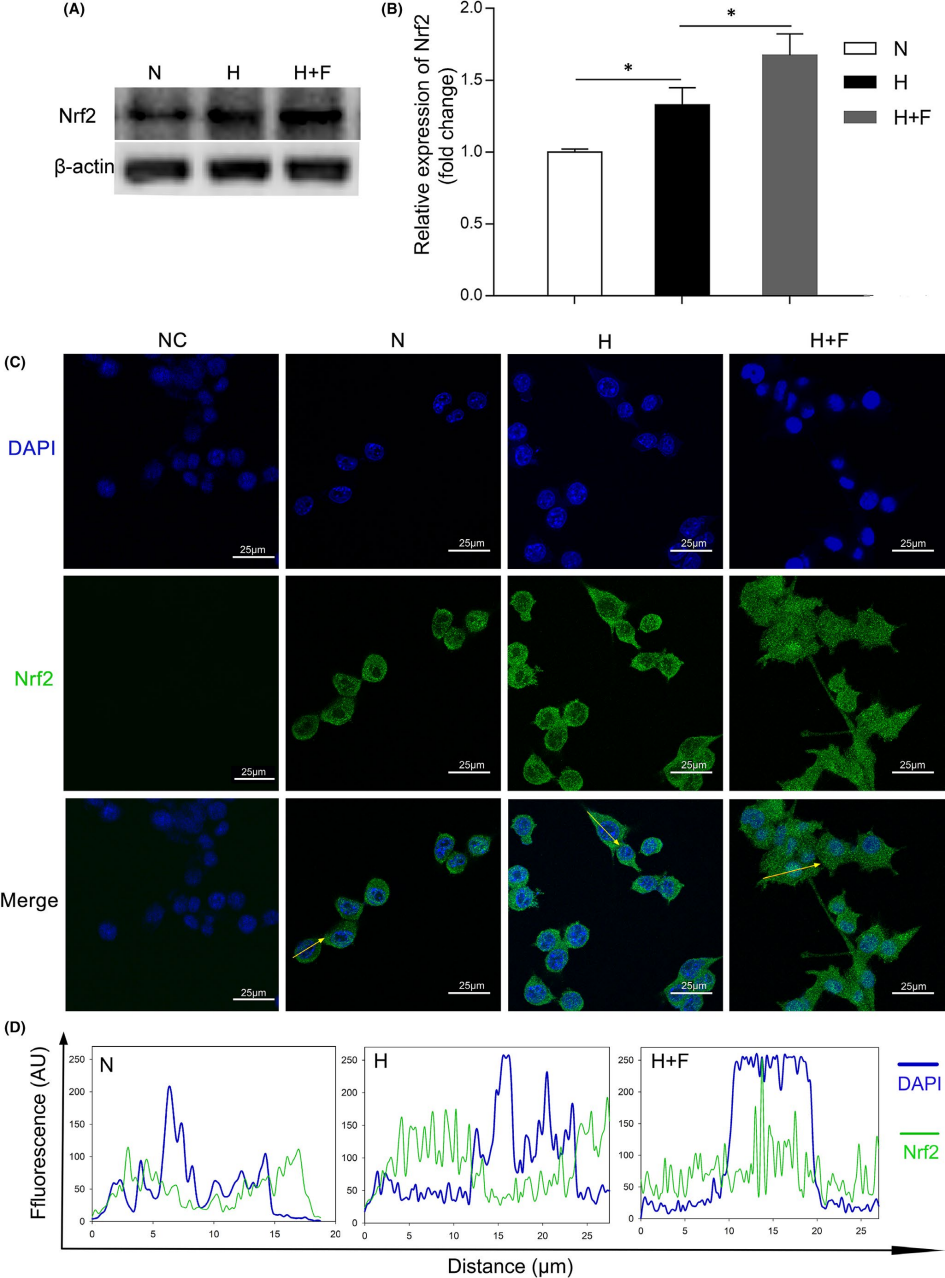
FKN enhanced the activation of Nrf2 in hypoxia‐induced microglia. (A and B) Western blot densitometry analysis of Nrf2 in hypoxia‐treated BV2 cells with or without FKN treatment. (C) Immunofluorescence of Nrf2 in hypoxia‐treated BV2 cells with or without FKN treatment. (D) The quantitation of the fluorescence intensity among three groups. The profiles along an ideal oriented line across a representative cell indicated by yellow arrows in [C] was quantitated by ImageJ software. Data were expressed as mean ± SE (*n* = 4 samples per group), and *p < 0.05 compared with hypoxia group). AU, Arbitrary Units. N, normal control; H, Hypoxia group; H+F, hypoxia‐treated BV2 cells co‐treated 5 ng/ml FKN. Scale bar: 25 μm

**FIGURE 8:**
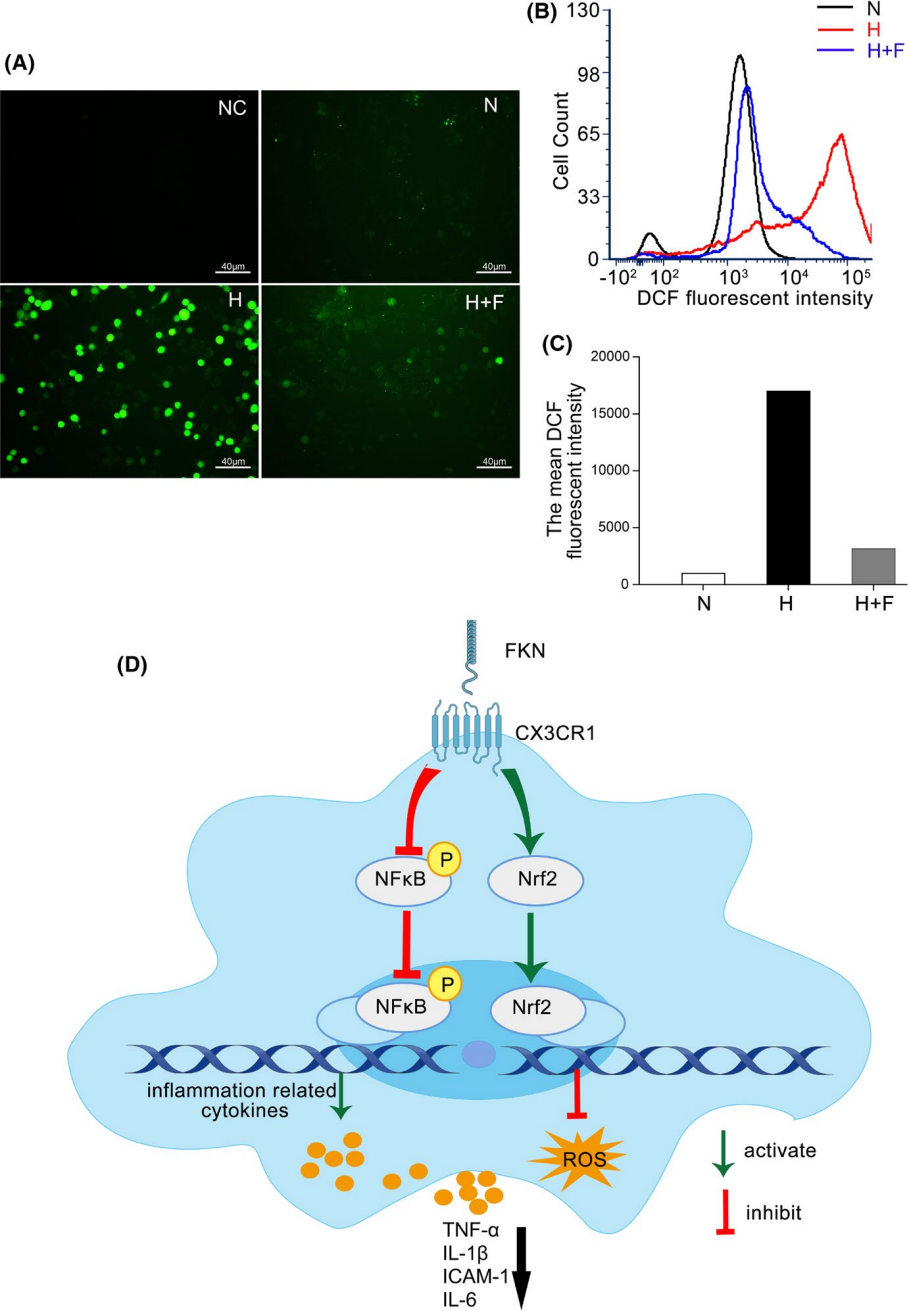
Detection of intracellular ROS level by DCF fluorescence intensity and schematic diagram showing FKN/CX3CR1 signalling pathway. DCF fluorescence of BV2 cells observed by fluorescence microscope among three groups. DCF fluorescence of BV2 cells among three groups was assayed with flow cytometry. Mean fluorescent intensity of DCF in BV2 cells in [B] was quantitated among three groups. The schematic diagram showing the FKN/CX3CR1 signalling pathway influenced by FKN treatment in activated microglia. N, normal control; H, hypoxic group; H+F, hypoxia‐treated BV2 cells co‐treated 5 ng/ml FKN. Scale bar: 40 μm

As shown in Figure 7A and Figure 7B, the Western blot densitometry analysis showed that, compared with normal control, Nrf2 protein expression was increased in hypoxia‐treated group, increased by 32.9% (*n* = 4, *p* = 0.036), which was further up‐regulated by 26.0% (*n* = 4, *p* = 0.047) after FKN treatment. To further characterize the activation of Nrf2, we detected the translocation of Nrf2 in BV2 cells by immunofluorescence. As shown in Figure 7C and Figure 7D, Nrf2 protein was mainly distributed in cytoplasm in normal control group and hypoxia group. However, FKN treatment promoted the translocation of Nrf2 from the cytoplasm to the nucleus. To see if Nrf2 translocation could enhance the clearance of ROS in microglia, we measured intracellular ROS level using both fluorescence microscope and flow cytometry in BV2 cells treated with DCF‐DA. The results showed that the level of intracellular ROS increased obviously in hypoxic group compared with that in normal control, which was largely dampened by FKN (Figure 8A). To quantify the ROS level, we conducted flow cytometry and found that ROS level in hypoxic group was about 17‐fold of that in normal control, which was decreased by 81.5% after FKN treatment (Figure 8B‐C).

## 4 DISCUSSION

A tight‐knit relationship between FKN/CX3CR1 signalling pathway and the complex network of neurons and glia has been reported in the central nervous system diseases, ocular diseases and other organ diseases.^11^, ^32^, ^33^, ^34^, ^35^, ^36^ In the current study, we focused on the possible relationship between retinal neurodegeneration and microglia activation, as well as the potential effect of FKN on microglia activation in DR. The data showed that exogenous FKN treatment could attenuate the activation of microglia in DR to decrease the expression of inflammation‐related cytokines via inhibiting NF‐κB and eliminate ROS production via activating Nrf2, which exerted its protective effect through CX3CR1.

Abnormal expression of FKN has been reported in many central nervous system diseases. For example, FKN expression declined in mouse model of Alzheimer's Disease (AD),^10^ in the cerebrospinal fluid of patients with AD,^12^ in patients with moderate/severe stroke,^13^ and in the brain of aged rats accompanied by an age‐related increase in microglial activation,^37^ whereas, FKN expression was reported to be up‐regulated under other conditions. For example, FKN expression increased in the cerebrospinal fluid and nucleus tractus solitaire, after 2 weeks of fructose feeding‐induced hypertension^34^ and in aqueous humour of Ins2Akita diabetic mice.^17^ The differential expressions of FKN under different conditions indicated its diverse roles in a certain disease. In our study, FKN was down‐regulated with diabetes progression accompanied by neuronal apoptosis in retinas of STZ‐induced diabetic rats and the *in vitro* study with glyoxal‐treated R28 cells indicated that the decreased FKN expression was at least partially due to the loss of retinal neurons in experimental DR (Figures 1, 2).

The regulation of FKN on microglia activation is dependent on its receptor CX3CR1. Previous studies showed CX3CR1 depletion accelerated microglial activation^10^ and enhanced the phagocytic capacity of microglia^38^ in mouse model of AD, indicating the inhibitory effect of CX3CR1 on microglia activation. FKN treatment could maintain microglia in a quiescent state, suggesting its neuroprotective effect via modulation of microglia in rodents with permanent focal cerebral ischemia^14^ and in rat model of Parkinson's disease.^15^ As for its effect in the ocular diseases, FKN/CX3CR1 signalling pathway was reported to exhibit beneficial effects,^9^, ^16^, ^17^, ^18^, ^20^ whereas, controversial studies also existed, showing FKN/CX3CR1 signalling participates in disease progression. For example, intravitreal injection of anti‐mouse FKN antibody could reduce the retinal angiogenesis in the oxygen‐induced retinopathy model,^39^ and CX3CR1 depletion could attenuate renal inflammation, renal fibrosis and renal injury in diabetic nephropathy model.^36^ Our results supported the idea that FKN treatment could reduce neuroinflammation by attenuating microglia activation in DR (Figure 3, 4, 5). These preliminary findings support that FKN/ CX3CR1 signalling pathway plays a protective role in experimental DR, which warrants further study with more focus on the neuroprotective and vascular protective effects of FKN.

The molecular mechanisms involved in FKN/CX3CR1 signalling pathway are very complex and still remain unknown despite the recent progress.^22^, ^40^, ^41^, ^42^ Both NF‐κB and Nrf2 signalling pathways were reported to be downstream of FKN/CX3CR1 and extensively studied.^22^, ^26^, ^31^, ^43^, ^44^ The relationships between NF‐κB suppression and anti‐inflammation exerted by FKN were reported in different animal models.^43^, ^44^ In the current study, this relationship was also confirmed in experimental DR (Figure 6), indicating that the anti‐inflammatory effect by FKN via suppression of NF‐κB activation might be universal in different neuroinflammatory diseases. Furthermore, prior studies noted the importance of Nrf2 in its anti‐oxidant and anti‐inflammatory effects in BV2 cells,^27^, ^45^, ^46^, ^47^ which was like downstream target of FKN.^22^, ^26^, ^31^ Our study also demonstrated that FKN treatment enhanced the Nrf2 activation and accelerated ROS elimination in hypoxia‐induced microglia (Figures 7 and 8). Nonetheless, sophisticated crosstalk between Nrf2 and NF‐ĸB signalling pathways remains completely unknown^28^, ^48^ and deserved further study. The limitation of the current study is lack of relevant inhibitors for proteins such as Nrf2, NF‐ĸB and soluble CX3CR1, to elucidate the upstream and downstream molecular events from FKN/CX3CR1 to the expressions of the inflammation‐related cytokines.

## 5 CONCLUSIONS

The present study demonstrated that the decreased FKN expression was associated with the retinal neuronal cell apoptosis and microglia activation in experimental DR. Exogenous FKN via intravitreal injection attenuated microglia activation and inflammation‐related cytokines in diabetic rat retina. Furthermore, FKN exerted its regulatory effect on microglia to reduce the neuroinflammation and ROS production through suppressing NF‐κB pathway and enhancing Nrf2 pathway (Figure 8D). Thus, FKN might be a potential candidate, which warrants further investigation for the treatment of DR and other neuroinflammatory diseases.

## CONFLICT OF INTEREST

The authors declare that they have no conflict of interest.

## AUTHOR CONTRIBUTIONS

**Mengmeng Jiang:** Conceptualization (lead); Data curation (lead); Formal analysis (lead); Investigation (lead); Methodology (lead); Validation (lead); Visualization (lead); Writing – original draft (lead). **Hai Xie:** Investigation (supporting); Methodology (supporting); Validation (supporting). **Chaoyang Zhang:** Conceptualization (supporting); Data curation (supporting); Investigation (supporting); Methodology (supporting). **Tianqin Wang:** Investigation (supporting); Methodology (supporting); Validation (supporting). **Haibin Tian:** Methodology (supporting); Supervision (supporting). **Lixia Lu:** Methodology (supporting); Supervision (supporting). **Jing‐Ying Xu:** Methodology (supporting); Supervision (supporting). **Guo‐Tong Xu:** Funding acquisition (equal); Project administration (equal); Resources (equal); Supervision (equal); Writing – review & editing (supporting). **Lin Liu:** Funding acquisition (equal); Project administration (equal); Resources (equal); Supervision (equal). **Jingfa Zhang:** Conceptualization (supporting); Funding acquisition (equal); Investigation (supporting); Project administration (equal); Resources (equal); Supervision (equal); Writing – review & editing (lead).

## Data Availability

Data available on request from the authors.
